# Supplementary material for: Prevalence and correlates of symptoms of depression, anxiety, and psychological distress among women of reproductive age with delayed conception in urban and peri-urban low to mid-socioeconomic neighborhoods of Delhi, India: A cross-sectional study
Source: PLoS One. 2025 Feb 5;20(2):e0315347. doi: 10.1371/journal.pone.0315347 (PMC11798463; doi:10.1371/journal.pone.0315347)
Supplement: S1 Table — (DOCX) [file pone.0315347.s001.docx]

**Supplementary Document**

**Prevalence and association of symptoms of depression, anxiety, and psychological distress among women of reproductive age with delayed conception in urban and peri-urban low to mid-socioeconomic neighborhoods of Delhi, India: A cross-sectional study.**

Barsha Gadapani Pathak,^1,4^ Gitau Mburu,^2^ Ndema Habib,^2^ Rita Kabra,^2^ Aiysha Malik,^3^ James Kiarie,^2^ Ranadip Chowdhury,^1^ Neeta Dhabhai,^1^ Sarmila Mazumder^1^

1 Society for Applied Studies, New Delhi, India

2 UNDP-UNFPA-UNICEF-WHO-World Bank Special Programme of Research, Development and Research Training in Human Reproduction (HRP) Department of Sexual and Reproductive Health and Research, World Health Organization, Geneva, Switzerland.

3 Department of Mental Health and Substance Use, World Health Organization, Geneva, Switzerland

4 Centre for International Health, Faculty of Medicine, University of Bergen, Norway

**Corresponding author:** Sarmila Mazumder, Society for Applied Studies, New Delhi, India Email: [sarmila.mazumder@sas.org.in](mailto:sarmila.mazumder@sas.org.in)

S1-Table 1: Mental health status of women assessed by PHQ-4

| **Mental Health History (*Over the last 2 weeks: Proportion of women*)** | **n (%)** |
| --- | --- |
| **Had little interest or pleasure in doing things** | |
| Not at all | 500 (32.68) |
| Several days | 767 (50.13) |
| More than half the days | 174 (11.37) |
| Nearly everyday | 89 (5.82) |
| **Feeling down, depressed, or hopeless** | |
| Not at all | 472 (30.85) |
| Several days | 779 (50.92) |
| More than half the days | 165 (10.78) |
| Nearly everyday | 114 (7.45) |
| **Feeling nervous, anxious or on edge** |  |
| Not at all | 701 (45.82) |
| Several days | 604 (39.48) |
| More than half the days | 155 (10.13) |
| Nearly everyday | 70 (4.58) |
| **Not being able to stop or control worrying** |  |
| Not at all | 815 (53.27) |
| Several days | 545 (35.62) |
| More than half the days | 123 (8.04) |
| Nearly everyday | 47 (3.07) |
| **PHQ 4 scores** |  |
| Mean (SD) | 3.20 (2.90) |
| Median (IQR) | 3(0-4) |

*PHQ: Patient Health Questionnaire
